# Chemical composition and nutritional properties of *Gastrodia elata* water extract and its effects in rats with streptozotocin–induced diabetic osteoporosis

**DOI:** 10.3389/fnut.2025.1591070

**Published:** 2025-06-03

**Authors:** Fanfan Jia, Sirui Kang, Xinxin Li, Yiheng Quan, Ruiqing Gao, Chen Chen

**Affiliations:** Shaanxi Province Key Laboratory of Bio-resources, Qinba Mountain Area Collaborative Innovation Center of Bioresources Comprehensive Development, Qinba State Key Laboratory of Biological Resources and Ecological Environment (Incubation), College of Biological Science and Engineering, Shaanxi University of Technology, Hanzhong, Shaanxi, China

**Keywords:** *Gastrodia elata* Blume, diabetes mellitus, osteoporosis, water extract of *Gastrodia elata*, osteoblast, osteoclast

## Abstract

**Introduction:**

*Gastrodia elata* Blume (*G. elata*) is an edible medicinal homologous plant with a long history of use. However, to date, there is no evidence of the nutritional components and anti-diabetic and osteoporotic effects of the water extract of *G. elata* (WGE).

**Methods:**

In this study, WGE was prepared and analyzed for moisture, ash, protein, lipid, mineral, amino acid, total phenolic, and flavonoid content. WGE was analyzed using ultra-high performance liquid chromatography coupled with quadrupole time-of-flight mass spectrometry (UPLC-Q-TOF/MS). Furthermore, the effect of WGE on streptozotocin-induced osteoporosis in diabetic rats was explored, along with an in-depth examination of the underlying mechanisms.

**Results:**

The results indicated that WGE reduced blood glucose, water, food intake, and body weight and improved Hyperglycemia and organ coefficients. WGE treatment noticeably increased bone mineral density (BMD), repaired bone morphology (BM), restored bone histomorphometric parameters, and ameliorated pathological pancreatic lesions in diabetic rats. It significantly elevated the activity of glutathione (GSH), superoxide dismutase (SOD), and catalase in liver tissues, osteoblast numbers, and the mRNA and protein expression of osteoprotegerin (OPG) and runt-related transcription factor 2 (Runx2). WGE treatment reduced serum inflammatory cytokine levels and the number of osteoclasts and bone marrow adipocytes.

**Conclusion:**

The protective effect of WGE against diabetic osteoporosis arises from several mechanisms: lowering blood glucose levels, inhibiting oxidative stress and inflammation, and modulating OPG/receptor activator of nuclear factor-κB ligand (RANKL) expression. Our results indicated that WGE could serve as a theoretical foundation for the treatment of diabetic osteoporosis.

## Graphical Abstract

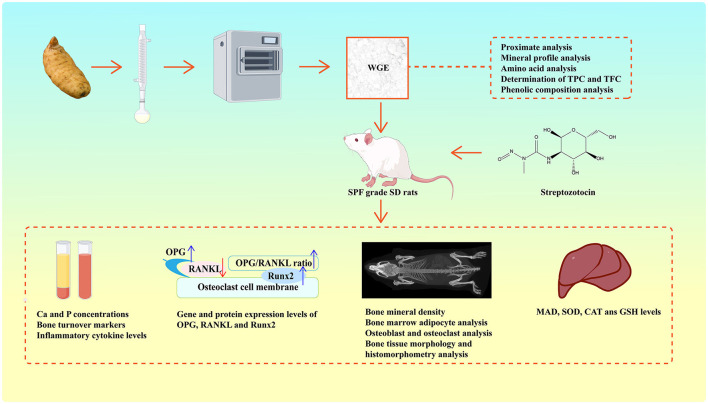

## 1 Introduction

Diabetes mellitus (DM) poses a serious risk to human health and is a global cause of death (1). According to the International Diabetes Federation, more than one in 10 adults worldwide currently has diabetes, and by 2045, 783 million adults in the 20–79 age group will have diabetes (2). DM-induced osteoporosis (DM-OS) is a chronic bone metabolic disorder characterized by altered bone microstructure, increased bone fragility, diminished bone mass per unit volume, and increased risk of fractures (3). The etiology of DM-OS is complex and involves various factors such as cellular abnormalities and matrix interactions (4). DM-OS, which is characterized by substantial disability and mortality, is a prominent focus of clinical research.

Various edible and medicinal homologous plants have been used to prevent DM-OS, and can effectively improve bone metabolism, increase bone mineral density (BMD), and improve clinical symptoms (5–7). A well-known example is *Gastrodia elata* Blume (*G. elata*), which has been used for thousands of years in China (8). Several chemical components of *G. elata* have been identified, including gastrodin, 4-hydroxybenzyl alcohol, parishin, and polysaccharides. *G. elata* has sedative, hypnotic, analgesic, immunity-enhancing, antitumour, antiviral, antioxidative, hypoglycaemic, antiradiation, antiaging, and neuroprotective effects. Furthermore, we previously demonstrated that gastrodin, a key constituent of *G. elata*, exerts protective effects against alcohol-induced liver injury (9). In China, it is a popular foodstuff, and *G. elata* is processed into different types of food, including wine, health tea, cakes, noodles, and other food additives (10).

Previous studies have shown that gastrodin can prevent dexamethasone-induced osteoporosis by improving osteoblast function (11). Gastrodin can prevent steroid-induced osteoporosis in rats and cell models, and promotes osteoblast formation by enhancing osteogenic differentiation and regulating Nrf2 (11). Furthermore, gastrodin can protect the cartilage by reducing inflammation, apoptosis, and matrix degradation (12). The results of study indicated that gastrodin is a promising candidate for osteoporosis prevention.

However, prior research has not addressed the protective effects of gastrodin or *G. elata* extracts on streptozotocin (STZ)-induced diabetic osteoporosis in rats or its associated mechanisms. Therefore, in this study, a water extract of *G. elata* (WGE) was prepared, and the components present in WGE were analyzed using ultra-high performance liquid chromatography with quadrupole time-of-flight mass spectrometry (UPLC-Q-TOF/MS). The total phenolic content (TPC), total flavonoid content (TFC), total carbohydrate content, and moisture, ash, protein, lipid, mineral, and amino acid content in WGE were analyzed in a constructed rat model of STZ-induced diabetic osteoporosis, and the improvement effect and possible mechanism of WGE on DM-OS were analyzed.

## 2 Results

### 2.1 Proximate, mineral, and amino acid components

We measured moisture, ash, protein, lipid, total polysaccharide, mineral, amino acid, and polyphenol content to determine the basic components of WGE (Table 1). The main components of WGE were total carbohydrates (38.24%) and proteins (9.21%), followed by moisture (4.60%), and lipid (4.01%). According to the results, WGE contained 16 types of amino acids, and the non-polar aspartic acid was the most abundant (7.47 mg/g dry weight (DW) of WGE). In WGE, the mineral content was the highest (2,547.75 mg/100 g DW of WGE), followed by the calcium content (222.47 mg/100 g DW of WGE).

**Table 1:** Proximate, minerals and amino acid compositions of WGE [dry weight (DW)].

**Component**	**Value**
**Proximate composition (g/100 g DW of WGE)**	
Moisture	4.60 ± 0.27
Ash	1.55 ± 0.11
Protein	9.21 ± 0.42
Liquid	4.01 ± 0.18
Total polysaccharide	38.24 ± 0.87
**Mineral composition (mg/100 g DWt of WGE)**	
Ca	222.47 ± 0.80
P	214.55 ± 1.94
K	2547.75 ± 7.71
Mn	2.15 ± 0.35
Zn	4.53 ± 0.10
Mg	144.56 ± 0.50
Na	51.06 ± 0.77
Cu	1.10 ± 0.09
Fe	4.51 ± 0.22
Se	0.01 ± 0.00
**Essential amino acid (mg/g DW of WGE)**	
Threonine	0.69 ± 0.05
Valine	0.86 ± 0.03
Methionine	0.32 ± 0.09
Isoleucine	0.34 ± 0.01
Leucine	0.59 ± 0.00
Phenylalanine	0.59 ± 0.05
Lysine	0.66 ± 0.07
Histidine	0.68 ± 0.03
Tyrosine	0.23 ± 0.04
Total essential amino acids	4.96 ± 0.82
**Nonessential amino acid (mg/g DW of WGE)**	
Proline	3.45 ± 0.11
Serine	1.31 ± 0.09
Glutamate	7.44 ± 0.14
Glycine	2.08 ± 0.10
Alanine	1.85 ± 0.09
Aspartic acid	7.47 ± 0.08
Arginine	0.88 ± 0.05
Cystine	ND
Total non-essential amino acids	24.48 ± 1.33
Total amino acids	29.44 ± 1.97
*Total polyphenols* (*mg GAE/g DW of WGE)*	47.04 ± 1.81
*Total flavonoids* (*mg RE/g DW of WGE)*	9.09 ± 0.98
**Polyphenol composition (mg/g DW of WGE)**	
Gastrodin	28.70 ±0.20
*P*-hydroxybenzyl alcohol	6.40 ±0.21
Orientin	1.62 ±0.01
Quercetin	0.49 ±0.00
Caffeic acid	0.33 ±0.00
Epicatechin	0.30 ±0.02
Catechin	0.15 ±0.00
Cinnamic acid	0.13 ±0.00
*P*-hydroxybenzoic acid	0.01 ±0.00

Results are the mean of three different determinations ± SD.

### 2.2 TPC, TFC, and phenolic compounds

In WGE, TPC was 47.04 mg GAE/g and TFC was 9.09 mg RE/g. The phenolic compounds detected in WGE included gastrodin, *p*-hydroxybenzyl alcohol, orientin, quercetin, caffeic acid, epicatechin, catechin, cinnamic acid, and *p*-hydroxybenzoic acid. Gastrodin content was the highest, at 28.70 mg/g (Table 1).

### 2.3 Characterization of components by UPLC-Q-TOF/MS

In WGE, 58 components were identified using UPLC-Q-TOF/MS, including polysaccharides, steroids, acids, and other compounds. Among them, phenolic compounds had the highest number of compounds with 48 compounds. Table 2 summarizes the retention times, molecular formulae, molecular weights, and molecular ions of the identified compounds. The structural formulae of the 58 constituents are provided in Supplementary Figure 2. The based peak intensity chromatograms are shown in the Supplementary Figure 1.

**Table 2:** The components profiling of WGE by UPLC-Q-TOF/MS.

**No**.	**Compounds**	**RT (min)**	***M*/*Z***	**Molecular formula**	**Molecular Weight**	**[*M* + *H*]**	**[*M*- *H*]**
1	6′-*O*-acetyl gastrodin	0.980	327.1077	C_15_H_20_O_8_	328.1158	–	√
2	Hentriacontanoic acid	0.989	465.4689	C_31_H_62_O_2_	466.4750	–	√
3	Citric acid	1.007	191.0194	C_6_H_8_O_7_	192.0270	–	√
4	Gastrodin B	1.041	363.1438	C_19_H_22_O_7_	362.1366	√	–
5	Uracil	1.259	113.0347	C_4_H_4_N_2_O_2_	112.0273	√	–
6	(–)-(*S/R S*)-γ-*L*-glutamyl-*L*-[*S*-(4-hydroxybenzyl)]cysteinyl glycine sulfoxide isomer I	5.003	430.1268	C_17_H_23_N_3_O_8_S	429.1206	√	–
7	(–)-(*S/R S*)-γ-*L*-glutamyl-*L*-[*S*-(4-hydroxybenzyl)]cysteinyl glycine sulfoxide isomer II	5.003	430.1268	C_17_H_23_N_3_O_8_S	429.1206	√	–
8	*N*-(4′-hydroxybenzyl)pyroglutamate	5.091	236.0922	C_12_H_13_NO_4_	235.0845	√	–
9	*N^6^*-(4-hydroxybenzyl)-adenosine	6.216	374.145	C_17_H_19_N_5_O_5_	373.1386	√	–
10	*N^6^*-(3-methoxyl-4-hydroxybenzyl) adenine riboside	6.444	404.1554	C_18_H_21_N_5_O_6_	403.1492	√	–
11	Adenosine	6.444	266.0884	C_10_H_13_N_5_O_4_	267.0968	–	√
12	Vanillin	6.504	153.054	C_8_H_8_O_3_	152.0473	√	–
13	5-[4′-(4″-hydroxybenzyl)-3′-hydroxybenzyloxymethyl]-furan-2-carbal-dehyde	6.601	337.1069	C_20_H_18_O_5_	338.1154	–	√
14	Parishin J	6.609	475.1493	C_20_H_26_O_13_	474.1713	√	–
15	5-(4-hydroxybenzyloxymethyl)-furan-2-carbaldehyde	6.627	233.0821	C_13_H_12_O_4_	232.0736	√	–
16	Ethyl(+)-(*S*)-{*N*-[4′-hydroxy-3′-(4″-hydroxybenzyl)benzyl]}pyroglutamate	6.645	368.1503	C_21_H_23_NO_5_	369.1576	–	√
17	Bis(4-hydroxybenzyl)ether mono-β-*L*-galactopyranoside	6.687	391.1390	C_20_H_24_O_8_	392.1417	–	√
18	2′/3′/4′/6′-*O*-(4″-hydroxybenzyl)gastrodin isomer I	6.687	391.1390	C_20_H_24_O_8_	392.1417	–	√
19	2′/3′/4′/6′-*O*-(4″-hydroxybenzyl)gastrodin isomer II	6.687	391.1390	C_20_H_24_O_8_	392.1417	–	√
20	2′/3′/4′/6′-*O*-(4″-hydroxybenzyl)gastrodin isomer III	6.687	391.1390	C_20_H_24_O_8_	392.1417	–	√
21	2′/3′/4′/6′-*O*-(4″-hydroxybenzyl)gastrodin isomer IV	6.687	391.1390	C_20_H_24_O_8_	392.1417	–	√
22	2′,6′/2′,7′/6′,7′-di-*O*-(p–hydroxybenzyl)gastrodin isomer I	6.714	499.1981	C_27_H_30_O_9_	498.1890	√	–
23	2′,6′/2′,7′/6′,7′-di-*O*-(p–hydroxybenzyl)gastrodin isomer II	6.714	499.1981	C_27_H_30_O_9_	498.1890	√	–
24	2′,6′/2′,7′/6′,7′-di-*O*-(p–hydroxybenzyl)gastrodin isomer III	6.714	499.1981	C_27_H_30_O_9_	498.1890	√	–
25	Gastrodigenin	6.775	125.0603	C_7_H_8_O_2_	124.0524	√	–
26	Hydroxymethyl-2-furancarboxaldehyde	6.775	125.0603	C_7_H_8_O_2_	124.0524	√	–
27	Parishin T/U isomer I	6.827	833.2491	C_39_H_46_O_20_	834.2528	–	√
28	Parishin T/U isomer II	6.827	833.2491	C_39_H_46_O_20_	834.2528	–	√
29	Parishin B/C isomer I	6.835	727.2068	C_32_H_40_O_19_	728.2164	–	√
30	Parishin B/C isomer II	6.835	727.2068	C_32_H_40_O_19_	728.2164	–	√
31	Parishin E/G isomer I	6.862	461.1268	C_19_H_24_O_13_	460.1217	√	–
32	Parishin E/G isomer II	6.862	461.1268	C_19_H_24_O_13_	460.1217	√	–
33	4-(4-hydroxyphenoxy)phenol	6.879	203.0715	C_12_H_10_O_3_	202.0630	√	–
34	Parishin N/O isomer I	6.932	489.1595	C_21_H_28_O_13_	488.1530	√	–
35	Parishin N/O isomer II	6.932	489.1595	C_21_H_28_O_13_	488.1530	√	–
36	Gastrodin	6.942	285.0941	C_13_H_18_O_7_	286.1053	–	√
37	4-hydroxybenzyl-β-*D*-glucopyranoside	6.942	285.0941	C_13_H_18_O_7_	286.1053	–	√
38	Hydroxymethylphenoxy-2-*O*-trans–cinnamoyl-β-*D*-glucoside	6.950	417.1559	C_22_H_24_O_8_	416.1471	√	–
39	1-*O*-(4-hydroxymethylphenoxy)-3/4/6-*O*-trans-cinnamoyl-β-*D*-glucoside isomer I	6.950	417.1559	C_22_H_24_O_8_	416.1471	√	–
40	1-*O*-(4-hydroxymethylphenoxy)-3/4/6-*O*-trans-cinnamoyl-β-*D*-glucoside isomer II	6.950	417.1559	C_22_H_24_O_8_	416.1471	√	–
41	1-*O*-(4-hydroxymethylphenoxy)-3/4/6-*O*-trans-cinnamoyl-β-*D*-glucoside isomer III	6.950	417.1559	C_22_H_24_O_8_	416.1471	√	–
42	Parishin	6.992	995.3031	C_45_H_56_O_25_	996.3111	–	√
43	Methyl (+)-(S)-2-hydroxy-3-[(4′-hydroxybenzyl) thio]propanoate	7.001	243.0682	C_11_H_14_O_4_S	242.0613	√	–
44	Trimethyl citrate	7.063	235.0818	C_9_H_14_O_7_	234.0740	√	–
45	Parishin D	7.098	403.1005	C_20_H_20_O_9_	404.1107	–	√
46	5-(hydroxymethyl)-furfural	7.264	127.0389	C_6_H_6_O_3_	126.0317	√	–
47	5-hydroxymethyl-2-furancarboxaldehyde	7.264	127.0389	C_6_H_6_O_3_	126.0317	√	–
48	4-hydroxybenzyl ethyl ether	7.350	153.0918	C_9_H_12_O_2_	152.0837	√	–
49	B-sitosterol glucoside	7.525	575.434	C_35_H_60_O_6_	576.4390	–	√
50	Gastrodin A	7.578	337.1443	C_21_H_20_O_4_	336.1362	√	–
51	Gastrol A/B isomer I	7.578	337.1443	C_21_H_20_O_4_	336.1362	√	–
52	Gastrol A/B isomer II	7.578	337.1443	C_21_H_20_O_4_	336.1362	√	–
53	Vitamin A	7.866	285.2195	C_20_H_30_O	286.2297	–	√
54	Parishin W	8.520	567.1735	C_26_H_30_O_14_	566.1636	√	–
55	Lacceroic acid	9.769	479.4828	C_32_H_64_O_2_	480.4906	–	√
56	Parishin L	10.616	1027.3291	C_46_H_58_O_26_	1026.3212	√	–
57	Parishin H/M isomer I	10.738	757.2217	C_33_H_42_O_20_	758.2269	–	√
58	Parishin H/M isomer II	10.738	757.2217	C_33_H_42_O_20_	758.2269	–	√

### 2.4 Effect of WGE on the general characteristics of DM rats

**Figure 1:**
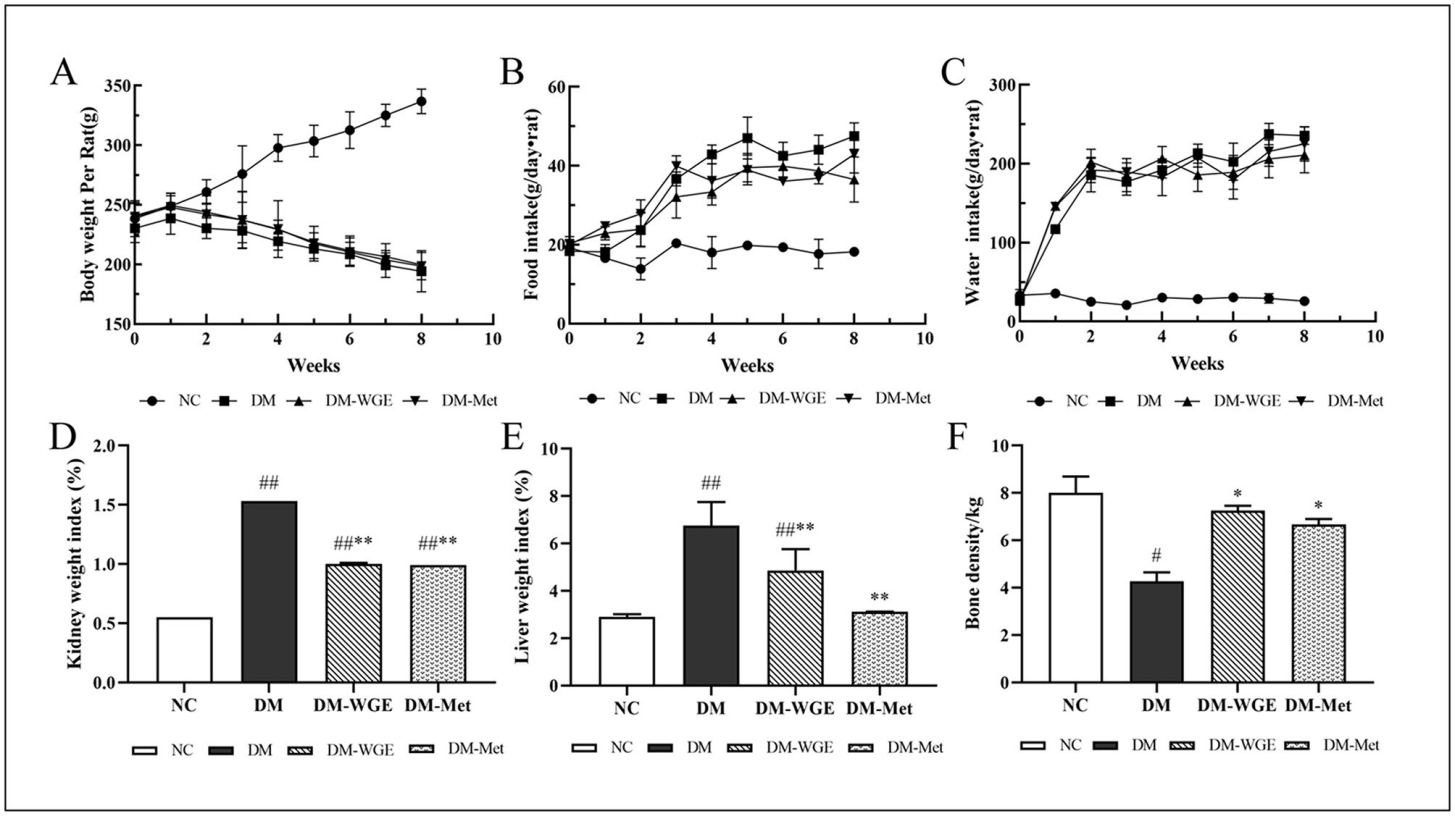
Effects of WGE on the general characteristics of STZ-induced DM rats. **(A–C)** Body weight, food intake, and water intake; **(D, E)** Kidney and liver weight index; **(F)** Bone mineral density. Values are the mean ± SD (*n* = 10). ^#^*p* < 0.05 and ^##^*p* < 0.01 vs. NC group; ^*^p < 0.05 and ^**^p < 0.01 vs. DM group.

During the eight-week experiment, body weight (BW) measurements revealed divergent trends among the groups of rats (Figure 1A). Rats in the normal control (NC) group exhibited a steady increase in BW, while those in the diabetes mellitus (DM) group showed a significant decrease (*p* < 0.05). Weight loss in the WGE-treated (DM-WGE) group was prevented by WGE treatment. Throughout the experiment, the food and water intake in the NC group remained stable. For the DM, DM-WGE, and Metformin-treated (DM-Met) groups, food and water intake after STZ injection was significantly elevated compared to the pre-injection levels (Figures 1B, C), with notable differences from the NC group (*p* < 0.05). The liver and kidney weight indices of the DM group were higher than those of the NC group (*p* < 0.01). After WGE treatment, the weight indices of the kidneys and liver in the DM-WGE group were significantly reduced (DM-WGE vs. DM, *p* < 0.01; Figures 1D, E).

Effect of WGE on BMD in DM rats. As depicted in Figure 1F, the BMD of rats in the DM group was reduced (NC vs. DM, *p* < 0.05); however, the BMD increased significantly after 8 weeks of WGE and metformin treatment (*p* < 0.05).

### 2.5 Effect of WGE on serum and liver biochemical parameters in DM rats

We measured blood glucose (BG), Ca, P, bone turnover markers, and inflammatory cytokine levels in the rat serum and antioxidant levels in the rat liver. The results are presented in Table 3. BG, alkaline phosphatase (ALP), receptor activator of nuclear factor-κB ligand (RANKL), interleukin-1β (IL-1β), interleukin-6 (IL-6), tumor necrosis factor-α (TNF-α), and malondialdehyde (MDA) levels in the DM group were elevated (DM vs. NC, *p* < 0.01). After the rats in the DM-WGE and DM-Met groups were treated with WGE and metformin, respectively, the BG, ALP, RANKL, TNF-α, IL-1β, IL-6, and MDA levels were reduced (DM-WGE and DM-Met vs. DM; *p* < 0.01). Levels of Ca, P, runt-related transcription factor 2 (Runx2), osteoprotegerin (OPG), osteocalcin, superoxide dismutase (SOD), catalase (CAT), and glutathione (GSH) were lower in the DM group than in the NC group (*p* < 0.01). Eight weeks after WGE treatment, Ca, P, Runx2, OPG, SOD, and GSH concentrations were reduced in the DM-WGE group (*p* < 0.01), while CAT content was elevated (*p* < 0.05). Nonetheless, the osteocalcin concentration did not increase in the DM-WGE group.

**Table 3:** Serum and liver biochemical parameters in STZ-induced DM rats.

**Parameter/group**	**Control**	**DM**	**DM-WGE**	**DM-Met**
Blood glucose (mmol/L)	5.76 ± 1.31	31.74 ± 5.37^##^	20.95 ± 5.64^##**^	21.06 ± 1.01^##**^
Ca (mg/dL)	9.69 ± 0.63	4.22 ± 0.21^##^	8.14 ± 0.92^#**^	8.35 ± 0.64^#**^
P (mg/dL)	7.59 ± 0.49	3.85 ± 0.37^##^	5.97 ± 0.41^##**^	6.13 ± 0.25^##**^
ALP (U/dL)	92.33 ± 7.12	198.45 ± 16.84^##^	110.27 ± 11.86^**^	127.26 ± 14.18^##**^
RANKL (ng/mL)	2.32 ± 0.19	7.82 ± 0.42^##^	2.74 ± 0.28^**^	3.21 ± 0.37^##**^
Runx2 (ng/mL)	12.97 ± 0.45	3.89 ± 0.31^##^	9.97 ± 0.33^##**^	7.42 ± 0.41^##**^
OPG (ng/mL)	9.12 ± 0.28	3.25 ± 0.19^##^	7.38 ± 0.41^##**^	6.97 ± 0.24^##**^
Osteocalcin (ng/mL)	22.38 ± 4.21	13.44 ± 2.10^##^	18.29 ± 2.25	15.99 ± 1.87
TNF-α (pg/mL)	30.54 ± 0.22	89.46 ± 6.89^##^	50.44 ± 3.59^##**^	58.62 ± 1.98^##**^
IL-1β (pg/mL)	61.05 ± 5.77	145.36 ± 12.37^##^	89.62 ± 8.43^##**^	97.54 ± 9.21^##**^
IL-6 (pg/mL)	45.88 ± 2.73	106.28 ± 5.77^##^	62.75 ± 8.26^##**^	73.46 ± 6.12^##**^
SOD (U/mg pro)	371.58 ± 89.23	142.08 ± 25.69^##^	285.67 ± 39.46^**^	234.35 ± 41.30^##^
CAT (U/mg pro)	274.56 ± 51.44	126.55 ± 30.12^##^	221.84 ± 26.22^*^	197.66 ± 30.19^#*^
GSH (μmol/g pro)	23.58 ± 2.13	6.38 ± 1.21^##^	17.55 ± 3.10^##**^	15.86 ± 2.25^##**^
MDA (nmol/mg pro)	5.28 ± 0.92	15.29 ± 0.38^##^	6.34 ± 0.40^#**^	6.77 ± 0.26^#**^

Results are the mean of three different determinations ± SD. ^#^p < 0.05 and ^##^p < 0.01 vs. NC group; ^*^p < 0.05 and ^**^p < 0.01 vs. DM group.

### 2.6 Histopathological alterations in the pancreas of DM rats

**Figure 2:**
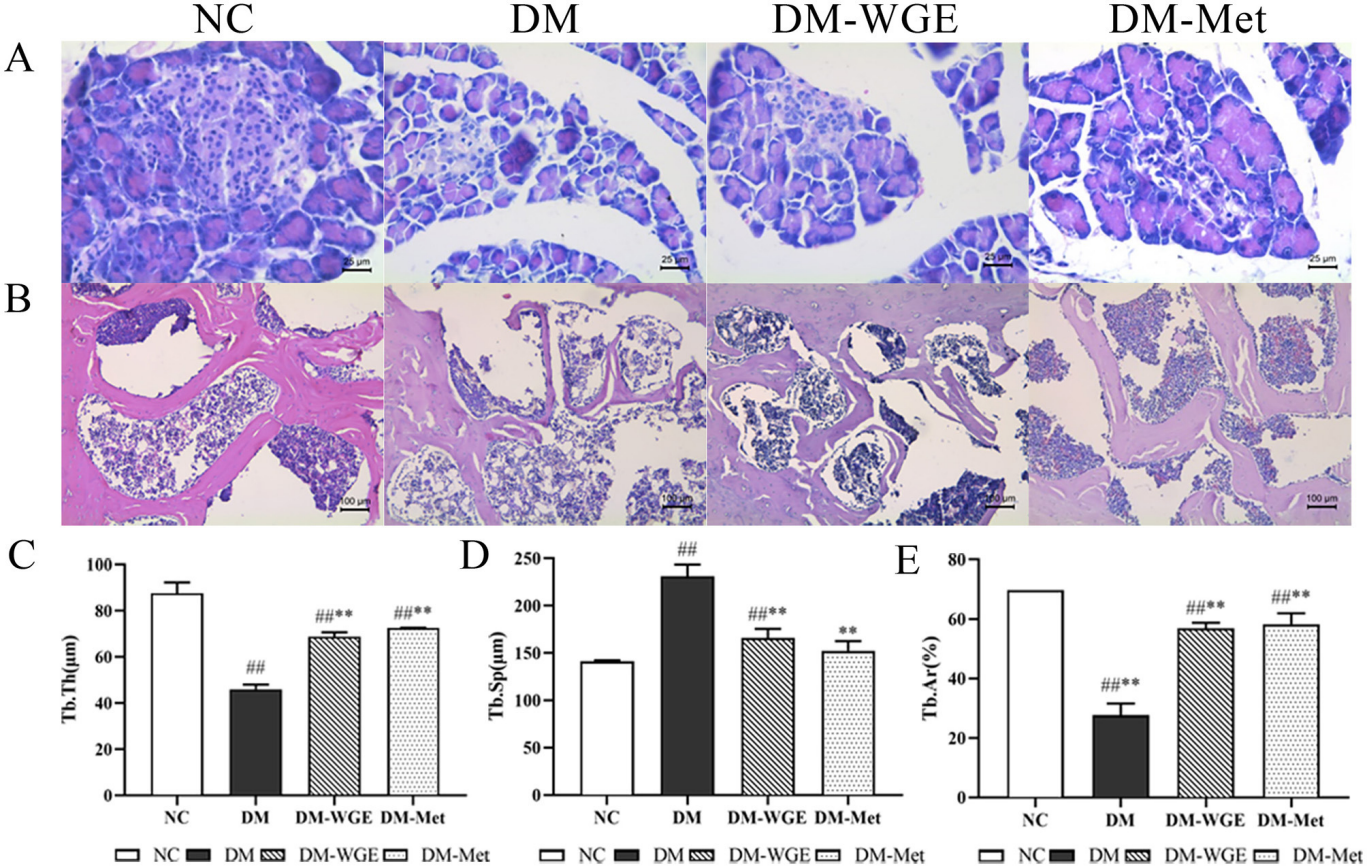
Effects of WGE on pancreatic pathology, bone morphology and bone histomorphometry parameters in STZ-induced DM rats. **(A)** Histopathological changes of pancreas, HE staining, magnification: 400×; **(B)** Femoral morphology of rats, HE staining, magnification: 200×; **(C–E)** Trabecular thickness (Tb·Th, μm), trabecular separation (Tb·Sp, μm), and trabecular area (Tb·Ar, %). Values are the mean ± SD (*n* = 10). ^##^*p* < 0.01 vs. NC group; ^**^p < 0.01 vs. DM group.

Compared with the NC group, the shape of islet cells in the DM group was irregular, and their volume and shape were smaller. In contrast, the DM-WGE group showed an increased islet cell count, more regular cell morphology, clearer boundaries, enriched cytoplasm, and larger cell volume compared with the DM group (Figure 2A).

### 2.7 Effects of WGE on bone histomorphometry parameters and morphology in DM rats

The bone trabeculae of the rats in the NC group formed a robust reticular network, which was tightly connected to each other, with complete morphology and clearly demarcated marrow cavities (Figure 2B). In contrast, the DM group exhibited sparse bone trabeculae with incomplete morphological structures, fractures, and irregular arrangements, along with significant expansion of the marrow cavity. In the DM-WGE group, the integrity of bone trabecular morphology and structure was significantly improved, the density of the trabecular bone was increased, the shape was regular, and the bone marrow cavity was significantly reduced. By analyzing trabecular thickness (Tb·Th), trabecular separation (Tb·Sp), and trabecular bone area (Tb·Ar) (Figures 2C–E), we found that the Tb·Th and Tb·Ar percentages in the DM group were significantly reduced, whereas the Tb·Sp percentage was significantly increased. After WGE treatment, the Tb·Th and Tb·Ar percentages were increased, whereas the Tb·Sp percentage was significantly reduced (*p* < 0.01). Compared with the DM group, femoral histopathology significantly improved after WGE treatment.

### 2.8 Effects of WGE on marrow adipocyte numbers in DM rats

**Figure 3:**
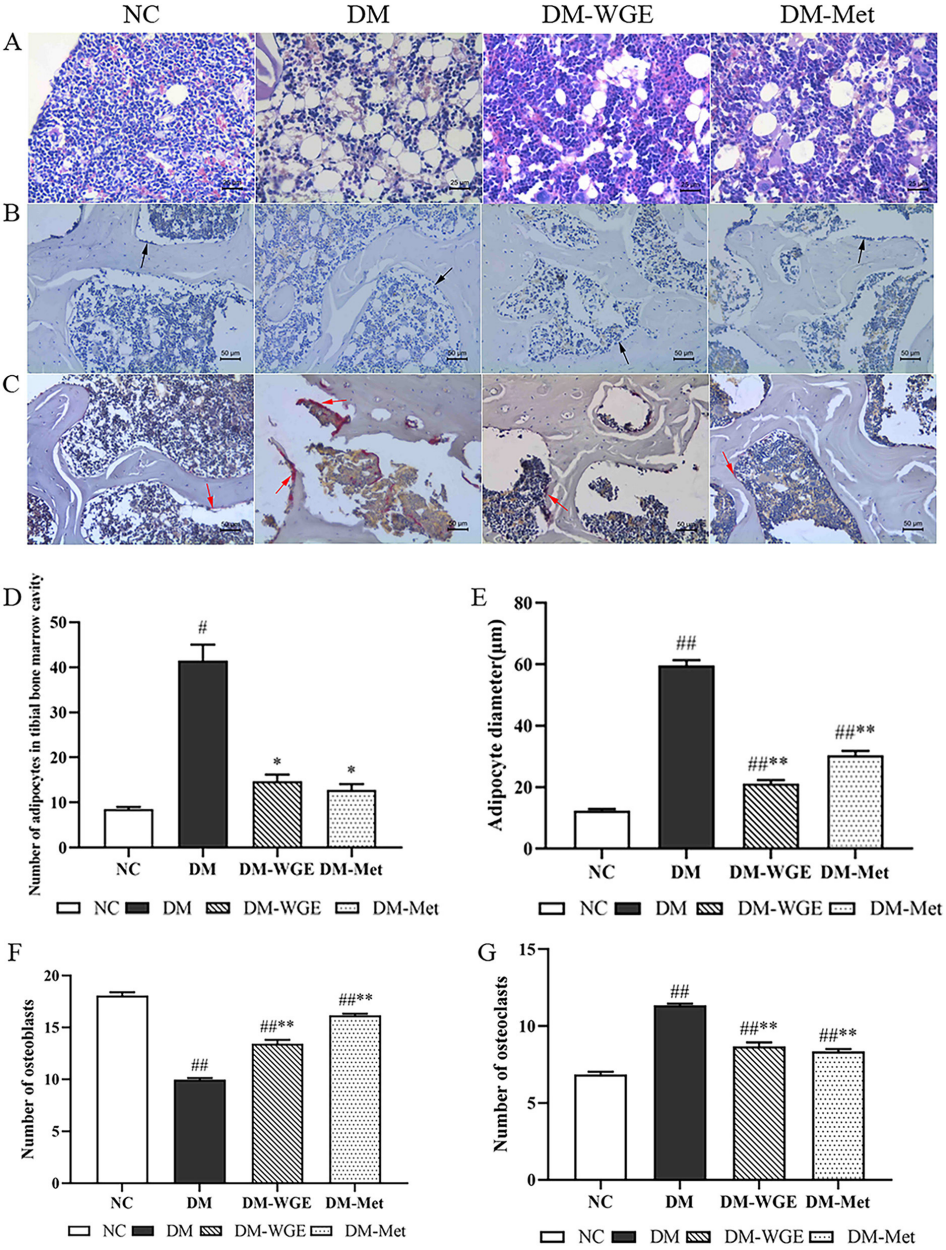
Effects of WGE on bone marrow adipocytes, osteoclasts and osteoblasts in STZ-induced DM rats. **(A)** Bone marrow adipocyte changes, HE staining, magnification: 200×; **(B)** Osteoblasts in femoral tissue, Ca–Co staining (200×); **(C)** Osteoclasts in femoral tissue, TRAP staining (200×); **(D)** Bone marrow adipocyte density of rats; **(E)** Bone marrow adipocyte diameter of rats; **(F)** Osteoblast numbers. **(G)** Osteoclast numbers. The black and red arrows in the picture indicate osteoblasts and osteoclasts, respectively. Values are the mean ± SD (*n* = 10). ^#^*p* < 0.05 and ^##^*p* < 0.01 vs. NC group; ^*^p < 0.05 and ^**^p < 0.01 vs. DM group.

In the NC group, tibial marrow adipocytes were relatively small, while the DM group exhibited numerous and enlarged marrow adipocytes (Figure 3A). Counting analysis revealed a noticeable increase in the marrow adipocyte count in the DM group (DM vs. NC, *p* < 0.05; Figure 3D), and the number of rat bone marrow adipocytes treated with WGE and metformin was reduced (*p* < 0.05). The diameter of marrow adipocytes in the DM group was increased (DM vs. NC, *p* < 0.01; Figure 3E), whereas that of marrow adipocytes in the tibia of WGE- and metformin-treated rats was significantly reduced (*p* < 0.01).

### 2.9 Effects of WGE on osteoblasts and osteoclasts in DM rats

Osteoblasts were mainly distributed around the bone trabeculae and arranged in a granular or short column shape with gray-black cytoplasm (Figure 3B). In femoral bone tissues, osteoclasts showed a deep purplish-red color after tartrate-resistant acid phosphatase (TRAP) staining (Figure 3C). The numbers of osteoblasts (Figure 3F) and osteoclasts (Figure 3G) decreased in the DM group (DM vs. NC, *p* < 0.01), whereas the number of osteoclasts significantly increased (*p* < 0.01). Osteoblast numbers significantly increased in the DM-WGE group after WGE treatment.

### 2.10 Effects of WGE on bone OPG, RANKL and Runx2 protein levels in DM rats

**Figure 4:**
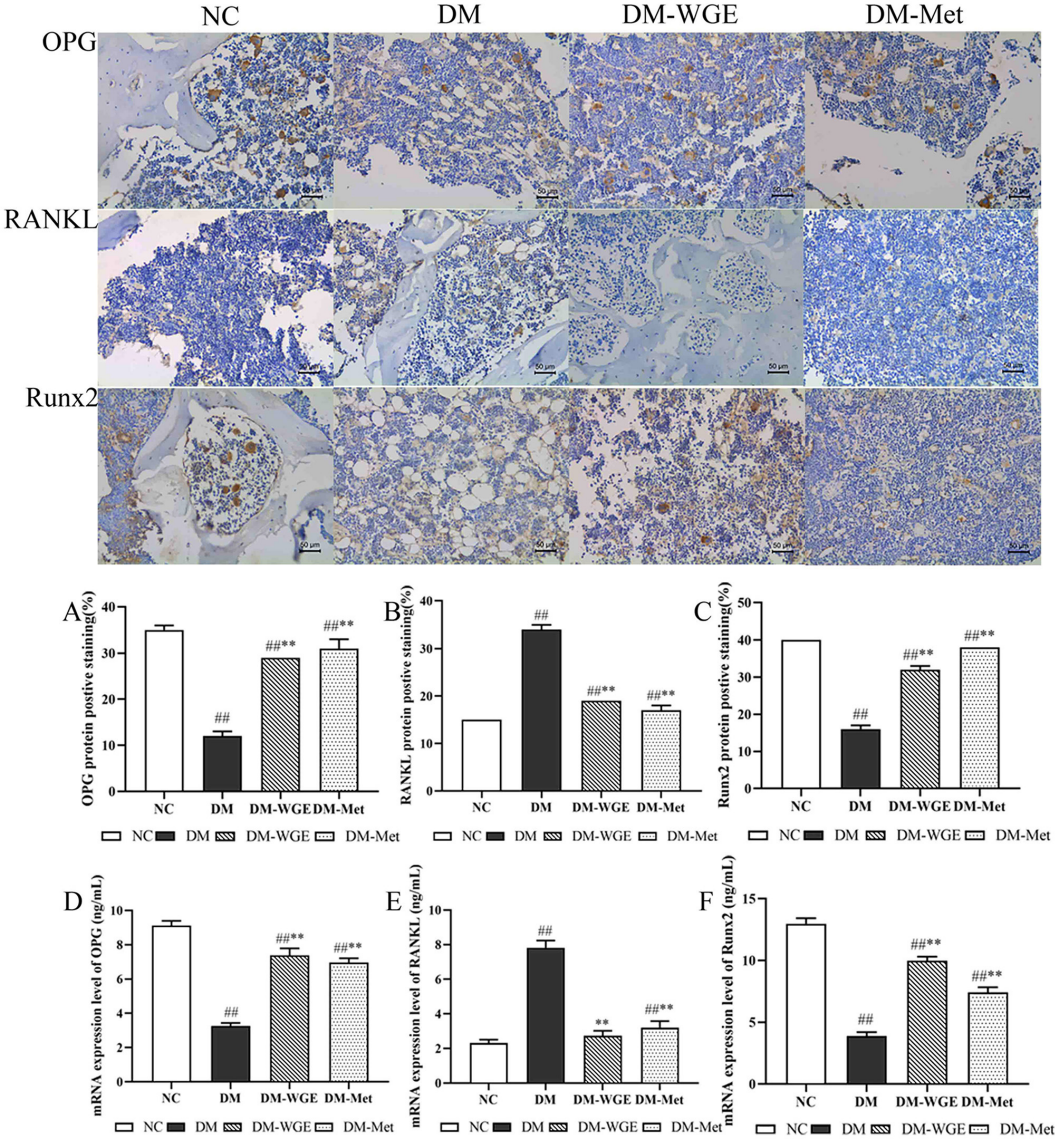
Effects of WGE on bone OPG, RANKL and Runx2 proteins and mRNA expression in STZ-induced DM rats. Immunohistochemical staining of OPG, RANKL and Runx2 proteins (Top) (400×); **(A–C)** Percentage of positive staining of OPG, RANKL, and Runx2 protein; **(D–F)** *OPG, RANKL*, and *Runx2* mRNA expression level. Values are the mean ± SD (*n* = 10). ^##^*p* < 0.01 vs. NC group; ^**^p < 0.01 vs. DM group.

We conducted immunohistochemical analysis to assess the protein expression of OPG, RANKL, and Runx2 in rat bone tissue, with the findings presented in Figure 4. OPG (Figure 4A) and Runx2 (Figure 4C) were highly expressed in the NC group, whereas RANKL was expressed at lower levels (Figure 4B). OPG and Runx2 protein expression levels in the DM group were significantly reduced (DM vs. NC, *p* < 0.01), whereas RANKL protein expression levels were elevated (*p* < 0.01). After treatment with WGE and metformin, the expression of OPG and Runx2 in the DM-WGE and DM-Met groups increased (DM-WGE and DM-Met vs. DM, *p* < 0.01), whereas that of RANKL protein was significantly reduced (DM-WGE and DM-Met vs. DM, *p* < 0.01).

### 2.11 Effects of WGE on bone OPG, RANKL, and Runx2 mRNA expression in DM rats

To determine whether WGE affected the expression of bone-related genes, we selected three bone cell genes for real-time PCR (qRT-PCR). Gene expression analysis suggested that *OPG* (Figure 4D) and *Runx2* (Figure 4F) were significantly downregulated in the DM group. Conversely, *RANKL* (Figure 4E) was upregulated in the DM group. After WGE treatment, the expression of *OPG* and *Runx2* remarkably increased (*p* < 0.01), whereas *RANKL* expression was suppressed.

## 3 Discussion

DM-OS, a chronic complication of diabetes in the skeletal system, is a major concern. It induces serious affects the normal life of patients (3). Currently, pharmaceuticals used to prevent osteoporosis mainly include bisphosphonates, parathyroid hormones, calcium, and selective estrogen receptor modulators. Although these chemical drugs have proven effective in treating osteoporosis, there are still problems with patient medication compliance, such as adverse reactions; thus, their long-term use is not recommended (13). Numerous edible and medicinal homologous plants have been used to treat osteoporosis, and have shown preventive and therapeutic effects (5–7). These plants have few side effects and have a variety of active ingredients for anti-osteoporotic agents, including flavonoids, polysaccharides and polyphenols (14). We have previously reported that salidroside from *Rhodiola rosea* L. exhibits anti-osteoporosis effects (15). One possible mechanism involves the prevention and treatment of osteoporosis through their antioxidant and anti-inflammatory effects (16, 17).

A recent study showed that WGE and its bioactive compounds exhibited both locomotor improvement and protective effects on dopaminergic neurons (18). The result of UPLC-Q-TOF/MS showed that WGE contained the highest number of phenolics among the 58 compounds, suggesting that phenolics are the main active components of WGE and may play a major role in the treatment of osteoporosis. Yam (*Dioscorea* spp.) is also an edible and medicinal plant in China, and like *G. elata*, also have health benefits. Padhan et al. determined the total polyphenol content to range from 2.19 to 9.62 mg/g in eight wild yams, much lower than the total polyphenol content of WGE (19), this further confirming our suspicions. Notably, polyphenolic compounds such as gastrodin, *p*-hydroxybenzyl alcohol, and orientin were detected in high abundance. Previous studies have demonstrated that both gastrodin and orientin exert anti-osteoporotic effects *in vitro* and *in vivo* (11, 20, 21). However, these effects have primarily been studied in isolation, and the combined or synergistic impact of these compounds as part of a whole extract, such as WGE, has not been previously reported. To further investigate WGE's anti-osteoporotic effects in diabetic rats, we evaluated its influence on OPG/RANKL expression. Compared to yam, the WGE polyphenols prepared by us has a higher polyphenol content, which is 4.89–21.48 times that of yam polyphenols. Therefore, the high content of polyphenols and flavonoids in WGE may be the main chemical component responsible for its anti-osteoporotic effects. The WGE prepared in this study contained various mineral elements and exhibited several regulatory advantages in DM rats. The calcium content in WGE is 222.47 mg/kg, indicating that it was a high-calcium food (the Chinese high-calcium standard is 150–500 mg/100 g in food). Therefore, we concluded that calcium-rich WGE improves the symptoms of diabetic osteoporosis.

Bone tissue exhibits a balanced process of continuous absorption, formation, and reconstruction, and osteoblasts and osteoclasts play important roles in maintaining this balance. Osteoclasts are one of the main functional cells in bone tissue resorption and are directly involved in the process, their activity directly reflects bone resorption ability. ALP is involved in bone formation and mineralization, and osteoblast differentiation is specifically mediated by ALP. Therefore, ALP activity was observed as osteoblast activity. Our results show that WGE treatment increased the number of osteoblasts and reduced the number of osteoclasts through ALP.

Reactive oxygen species (ROS) are products of aerobic metabolism and are cleared by an organism's antioxidant system. However, when the body lacks sufficient antioxidant capacity to resist ROS, oxidative stress occurs, leading to cellular and tissue damage. Under such stress, both the activity and quantity of osteoblasts may decrease, accelerating bone loss and contributing to osteoporosis (22). In this study WGE increased SOD, CAT, and GSH activities and inhibited the level of MDA in lipid peroxidation products; that is, it relieved osteoporosis in DM rats through antioxidant pathways. These findings align with those of previous studies that demonstrated the protective effects of gastrodin against osteoporosis by reducing ROS (23).

The amino groups of proteins, lipids, nucleic acids, and reducing sugars react non-enzymatically in physiological environments to produce advanced glycation end-products (AGEs). Long-term Hyperglycemia causes the accumulation of several AGEs in the body. AGEs can act on various cells, combine with cell-surface receptors, produce various cytokines (IL-1β, IL-6, and TNF-α), contribute to the differentiation and maturation of osteoclasts, osteoclast activity, and accelerate bone resorption. A previous study has documented that gastrodin can attenuates lipopolysaccharide-induced inflammation. This effect is associated with decreased levels of inflammatory factors (24). Furthermore, studies have reported that gastrodin can decrease the production of inflammatory mediators, such as IL-6 and TNF-α (12). TNF-α can enhance osteoclast differentiation and maturation, leading to an increased osteoclast count and reduced bone mineralization. In addition, TNF-α can inhibit osteoblast function, reduce ALP activity, and reduce calcium deposition in bone tissue. Long-term Hyperglycemia promotes the synthesis and secretion of TNF-α in diabetes, thus enhancing osteoclast activity and accelerating bone loss, which is an important pathogenesis of diabetic osteoporosis (3). In this study, WGE reversed the recovery of pancreatic islet cells in DM rats, inhibited blood sugar levels, and reduced concentrations of IL-1β, IL-6, and TNF-α.

The OPG/RANK/RANKL signaling pathway is a key pathway in bone reconstruction. OPG, an activator of RANKL, binds to RANKL during bone turnover, blocks RANKL/RANK signal transduction, and inhibits osteoclast differentiation and maturation. The combination of RANKL and RANK triggers activation of the RANK pathway, increases the development and activation of osteoclast precursors, maintains osteoclast activity, and inhibits apoptosis (25). Previous research has suggested that the mRNA levels of osteogenic genes, including *Runx2*, osterix, bone morphogenetic protein-2, and osteocalcin, are elevated in groups pretreated with gastrodin (11). *Runx2* can directly bind to the promoter region of the RANKL and activate its transcription, inducing osteoblast differentiation and increases the number of immature osteoblasts that form immature bone tissue. We measured Runx2 protein and mRNA expression levels in DM rats and showed that they were elevated in DM rats after STZ injection and decreased after WGE treatment. At the same time, following WGE treatment, the OPG protein level in rat bone tissue increased, whereas the RANKL protein level decreased; the mRNA and protein expression levels showed similar trends. Therefore, WGE ameliorates diabetic osteoporosis in rats by reducing the expression of *Runx2* and OPG and inhibiting the activation of the OPG/RANK/RANKL pathway.

## 4 Materials and methods

### 4.1 Chemicals

Folin–Ciocalteu reagent (CAS: 12111-13-6), Folin–Dennis reagent (CAS: F-0443542), STZ (CAS: 18883-66-4), metformin (CAS: 657-24-9), Gomori's calcium-cobalt (Ca-Co) staining kit and TRAP staining kit were all provided by Sigma–Aldrich (Steinheim, Germany). Standard for monomer phenolic compounds (Shidande, Shanghai, CHN). ALP, RANKL, Runx2, OPG, osteocalcin, TNF-α, IL-1β and IL-6 enzyme-linked immunosorbent assay (ELISA) kits (TW-Reageng, Shanghai, CHN). CAT, SOD, MDA and GSH kits (Jiancheng, Nanjing, CHN). The OPG (A00863) antibodies were bought from Wuhan Boster Biological Technology., LTD (Wuhan, CHN). The RANKL (YP-Ab-14022), Runx2 (SYP-R1767) antibodies were bought from Uping Bio technology Co., Ltd (Shenzhen, CHN). Total RNA rapid extraction kit, cDNA reverse transcription kit, SYBR-Green Mix and PCR kit (Takara, Beijing, CHN).

### 4.2 Raw and processed plant samples

Fresh *G. elata* rhizomes were collected at harvest in November 2022 from Lueyang County, Shaanxi Province, China (33°31′N, 106°13′E) and identified by Dr. Yong Wang, a botanist at the Shaanxi Polytechnic University. The collected rhizomes were washed with tap water, steamed, cut into slices, and dried at 60°C. Dried plant samples were ground in a laboratory mill and sieved (80-mesh).

### 4.3 Preparation of *G. elata* plant extracts

*G. elata* powder was accurately weighed and placed in a round bottom flask. Distilled water was added (sample:solvent, *m*:*v*, 1:37), the mixture was subjected to reflux extraction (100°C, 2 h) and vacuum filtration; the supernatant was collected, and the extraction was repeated twice. The extracts were cooled to room temperature, centrifuged (4°C, 10,000 rpm, 10 min), decolorized using activated carbon, and freeze-dried to obtain a dry powder. The water extract of *G. elata* was named WGE and maintained at −20°C in the dark until further chemical and bioactivity analyses. The WGE extraction yield was 25.10% (relative to the DW of *G. elata*).

### 4.4 Nutritional assessment

#### 4.4.1 Proximate analysis

Freeze-dried WGE was analyzed for moisture, ash, protein, and lipid content following the AOAC testing methods (26). The moisture content was measured by heating WGE at 105°C for 12 h until a constant weight was achieved, and the ash content was measured by weighing the residual gained after incineration at 550°C (24 h). The micro-Kjeldahl method (nitrogen content of the samples × 6.25) was used to determine crude protein content, the Soxhlet extraction method was used to determine crude lipid content, and total carbohydrate content was measured using the phenol-sulfuric acid method (27).

#### 4.4.2 Mineral profile analysis

Ca, P, K, Mn, Zn, Mg, Na, Cu, Fe, and Se mineral contents in WGE were determined using inductively coupled plasma mass spectrometry.

#### 4.4.3 Amino acid analysis

The amino acid composition of the WGE was quantified using a high-performance amino acid analyzer (Biochrom, Cambridge, UK) after acid hydrolysis (28). The WGE was hydrolyzed with 6 mol/L hydrochloric acid in a sealed tube at 110°C in an oven for 24 h. The hydrolysate was then added to a citrate buffer solution (pH 2.2). The hydrolysate was filtered through a membrane filter (0.22 μm) and then added to an amino acid analyzer.

#### 4.4.4 Determination of TPC and TFC

TPC and TFC were determined using Folin–Ciocalteu assays and the aluminum chloride method, respectively, as described in our previous work (29). The results were expressed as equivalents of gallic acid (mg GAE/g) for TPC and rutin (mg RE/g) for TFC.

#### 4.4.5 Phenolic composition analysis

The phenolic composition of WGE was determined using reverse phase-high-performance liquid chromatography (Dionex UltiMate 3000, Waltham, MA, USA) coupled with a diode array detector (DAD-3000RS) and an Inertsil WondaSil C_18_ analytical column (250 mm × 4.6 mm, 5 μm), as previously described (29).

#### 4.4.6 UPLC-Q-TOF/MS analysis

WGE components were analyzed using a Waters Acquity™ UPLC system (Waters Corporation, Milford, MA, USA) coupled with a Synapt G2 mass spectrometer (Waters Corporation) equipped with an electrospray ion source, based on methods previously established (29). The mass of the product ion scan ranged from 50 m/z to 1,500 (m/z).

### 4.5 *In vivo* animal model study

#### 4.5.1 Animal models

Forty specific pathogen-free Sprague-Dawley rats (male, 8 weeks old, 225–245 g) were purchased from the Dashuo Laboratory Animal Co., Ltd. (License No. SCXK (Sichuan) 2015-030). These rats lived in a purified environment at 22 ± 2°C under a relative humidity of 50–70% and an alternating 12 h light–dark cycle. Rats were fed a standard diet and had unrestricted access to food and water. All protocols in this study were approved by the Animal Ethics Committee of Shaanxi University of Technology (2022-07). The health status of the experimental animals, including weight, food and water intake, activity, and fur condition, was regularly monitored.

#### 4.5.2 Animal experimental design

The STZ-induced diabetic osteoporosis model was established as previously described (30). After adaptation for seven days, the NC group comprised 10 randomly assigned rats, and the remaining rats were intraperitoneally injected with STZ (60 mg/kg), a procedure that lasted for 3 days. Three days after STZ injection, rats were identified as DM rats if they had a random BG concentration ≥16.7 mmol/L. Based on our previous research (31), forty rats were divided into four groups, each comprising ten rats, as follows: (1) NC group: normal rats were administered 0.9% saline; (2) DM model group: DM rats received 0.9% saline; (3) DM-WGE group: DM rats received WGE at the dosage level of 2 g/kg BW. WGE doses were selected based on preliminary experiments conducted in our laboratory; (4) DM-Met group: DM rats received metformin 200 mg/kg BW. The metformin dosage was set according to the study by Ajiboye et al. (32). The four treatments were administered orally once daily for 8 weeks. At the conclusion of the experiment, all rats that fasted overnight were euthanized using carbon dioxide (CO_2_) at a chamber displacement rate of 10–30% per min, and their suffering was minimized to the maximum possible extent. For biochemical assays, blood was left to coagulate, and serum was obtained. Pancreas, kidneys, liver, and bones were collected from each animal. The calculated organ weight index is expressed as organ weight/body weight × 100. The pancreas and bones were fixed in 4% paraformaldehyde and maintained at −80°C for further use.

#### 4.5.3 Determination of bone mineral density

According to our previously proposed method (30), we dissected the lumbar vertebrae (L1–L4) and left femur obtained from rats with the soft tissues removed, and measured BMD using a dual-energy X-ray absorptiometry scanning system (GE Medical Systems, Madison, WI, USA).

#### 4.5.4 Determination of biochemical parameters in plasma and liver

Serum concentrations of Ca and P were determined using atomic absorption spectrometry (PerkinElmer, MA, USA), as previously described (30). We assayed ALP, RANKL, Runx2, OPG, osteocalcin, IL-1β, IL-6 and TNF-α using ELISA kits. Liver MDA, SOD, CAT, GSH levels were measured using appropriate kits. The absorbance of MDA, SOD, CAT, and GSH was measured at 532, 550, 405, and 420 nm, respectively, using a Bio-Tek Epoch microplate reader (BioTek Laboratories, Winooski, VT, USA).

#### 4.5.5 Pancreas histopathology

Fresh pancreatic tissues were fixed in 4% paraformaldehyde for 24 h, dehydrated, paraffin-embedded, and sectioned (5 μm) using a Leica slicer. Sections were stained with haematoxylin and eosin (HE) and sealed. Finally, the pathological injuries were examined using a ZEISS Axioscope 5 microscope (Carl Zeiss Meditec Inc., Dublin, CA, USA).

#### 4.5.6 Bone tissue morphology and histomorphometry analysis

Bone tissue morphology and histomorphometry were assessed using a previously described method (33). Briefly, femur tissue was fixed with 4% paraformaldehyde (24 h). Next, 10% ethylenediaminetetraacetic acid (EDTA) was added to the bone tissue, decalcified for 28 days, and passed through a gradient of ethanol (70, 80, 90, 95, and 100%). The sections were dewaxed, soaked in water, and stained with HE. Femur morphology and histomorphometry were examined using a micrograph system (Leica Biosystems, Wetzlar, Germany). Total tissue area (T·Ar), Tb·Ar, Tb·Th, and Tb·Sp were measured using Image Pro Plus (Media Cybernetics Inc., San Diego, CA, USA).

#### 4.5.7 Tibia bone marrow adipocyte analysis

The tibial bone was fixed in 4% paraformaldehyde (4°C, 24 h), decalcified with 10% EDTA for 28 days, embedded in paraffin wax, sectioned (5 μm), and stained with HE. Using a microphotography system (Leica Biosystems, Wetzlar, Germany) and Image-Pro Plus to measure the mean adipocyte diameter (μm) and number (number/mm^2^) in the tibial bone marrow.

#### 4.5.8 Osteoblast and osteoclast staining and analysis

Ca-Co and TRAP staining kits (Sigma-Aldrich, Steinheim, Germany) were used to stain the osteoblasts and osteoclasts. The morphology of osteoblasts and osteoclasts in the femoral head was observed under a microscope (Carl Zeiss Meditec Inc., Dublin, CA, USA). Five non-overlapping fields of view (×400) were randomly selected on each slide to accurately quantify the number of osteoblasts and osteoclasts in each field of view.

#### 4.5.9 Immunohistochemical staining and analysis

The protein expression of OPG, RANKL, and Runx2 in the bone tissue was examined using immunohistochemistry (34). Femoral sections were incubated in 1% Triton X-100 (25°C, 30 min) and citric acid solution (90°C, 60 min). The sections were rinsed three times with phosphate-buffered saline-Tween (PBS-T), blocked with 3% bovine serum albumin (25°C, 15 min), and then incubated in primary antibody solution (1:200, 37°C, 90 min). The cells were incubated with secondary antibody solution (1:250, 37°C, 120 min) and then washed with PBS-T. Nuclei were counterstained with haematoxylin after DAB staining. Areas showing positive staining were analyzed using Image J for percentage quantification.

#### 4.5.10 Quantitative qRT-PCR

After total RNA extraction from bone tissue, cDNA was obtained by reverse transcription. qRT-PCR was used to detect the mRNA expression of *OPG, RANKL*, and *Runx2* in bone tissue by referring to our previously proposed method, with GAPDH serving as an internal control (34). The 2^−ΔΔCt^ method was used to analyze the qRT-PCR data and the primer sequences used in this study are listed in the Supplementary Table 1.

### 4.6 Statistical analysis

All experiments were conducted in three independent parallel phases, and the results are expressed as mean ± standard deviation. The results were statistically analyzed using SPSS 26.0 (IBM Corporation, Armonk, NY, USA), and one-way analysis of variance followed by Tukey's multiple comparison *post-hoc* test was performed; *p* < 0.05 indicates statistical significance.

## 5 Conclusions

This study revealed that WGE is rich in nutrients, especially polyphenols, which act as the primary anti-osteoporotic chemical components. WGE notably reduced BG, water intake, food intake, and BW loss and improved Hyperglycemia and renal and liver organ coefficients in DM rats. WGE treatment noticeably increased bone mineral density, repaired bone morphology, restored bone histomorphometric parameters, and ameliorated pathological pancreatic lesions in DM rats. In addition, WGE significantly increased SOD, CAT, and GSH activities in liver tissues, osteoblast numbers, and the mRNA and protein expression of OPG and Runx2, while decreasing serum inflammatory cytokine levels, the number of bone marrow adipocytes, osteoclast numbers, and RANKL mRNA and protein expression levels. Our results suggest that WGE may provide a scientific foundation for the treatment of diabetic osteoporosis. Despite these promising findings, several limitations remain. Variations in the source material and processing methods may contribute to differences in outcomes, and the study has not yet been validated in human. To support the scientific and rational use of WGE, future research should incorporate samples from diverse sources and utilize various processing techniques. Additionally, the pharmacological effects of WGE should be validated across multiple animal models of osteoporosis and subsequently through clinical trials in humans.

## Data Availability

The original contributions presented in the study are included in the article/Supplementary material, further inquiries can be directed to the corresponding author.
