# Supplementary material for: Chemical composition and nutritional properties of Gastrodia elata water extract and its effects in rats with streptozotocin–induced diabetic osteoporosis
Source: Front Nutr. 2025 Jun 3;12:1591070. doi: 10.3389/fnut.2025.1591070 (PMC12170316; doi:10.3389/fnut.2025.1591070)
Supplement: Supplementary file 1 [file Data_Sheet_1.docx]

Supplementary Material

# Supplementary Figures and Tables

## Supplementary Figures

##
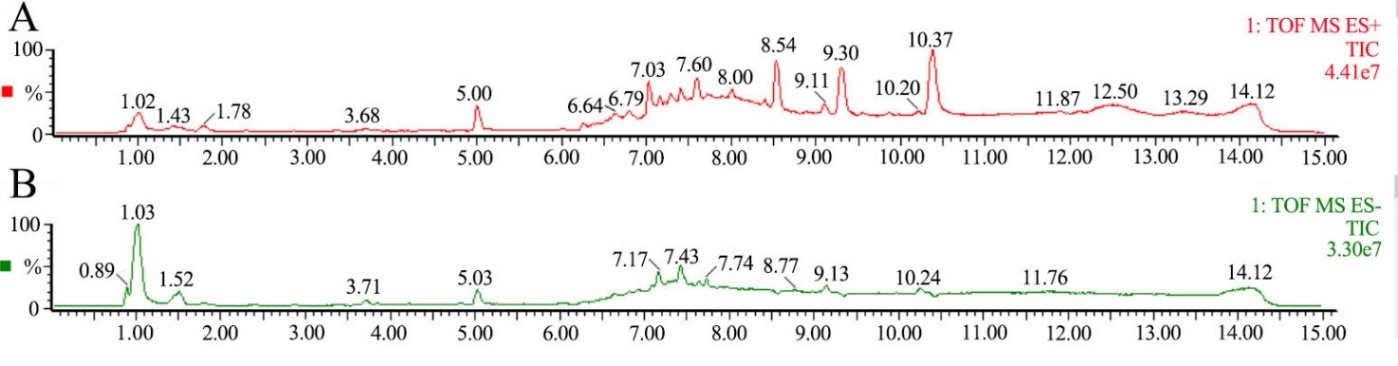


**Supplementary Figure S1.** BPI (base peak intensity) diagram of water extract of *Gastrodia elata* (WGE)*.* (A) Positive ion BPI (base peak intensity) diagram of *Gastrodia elata*; (B) Negative ion BPI (base peak intensity) diagram of water extract of *Gastrodia elata* (WGE)*.*


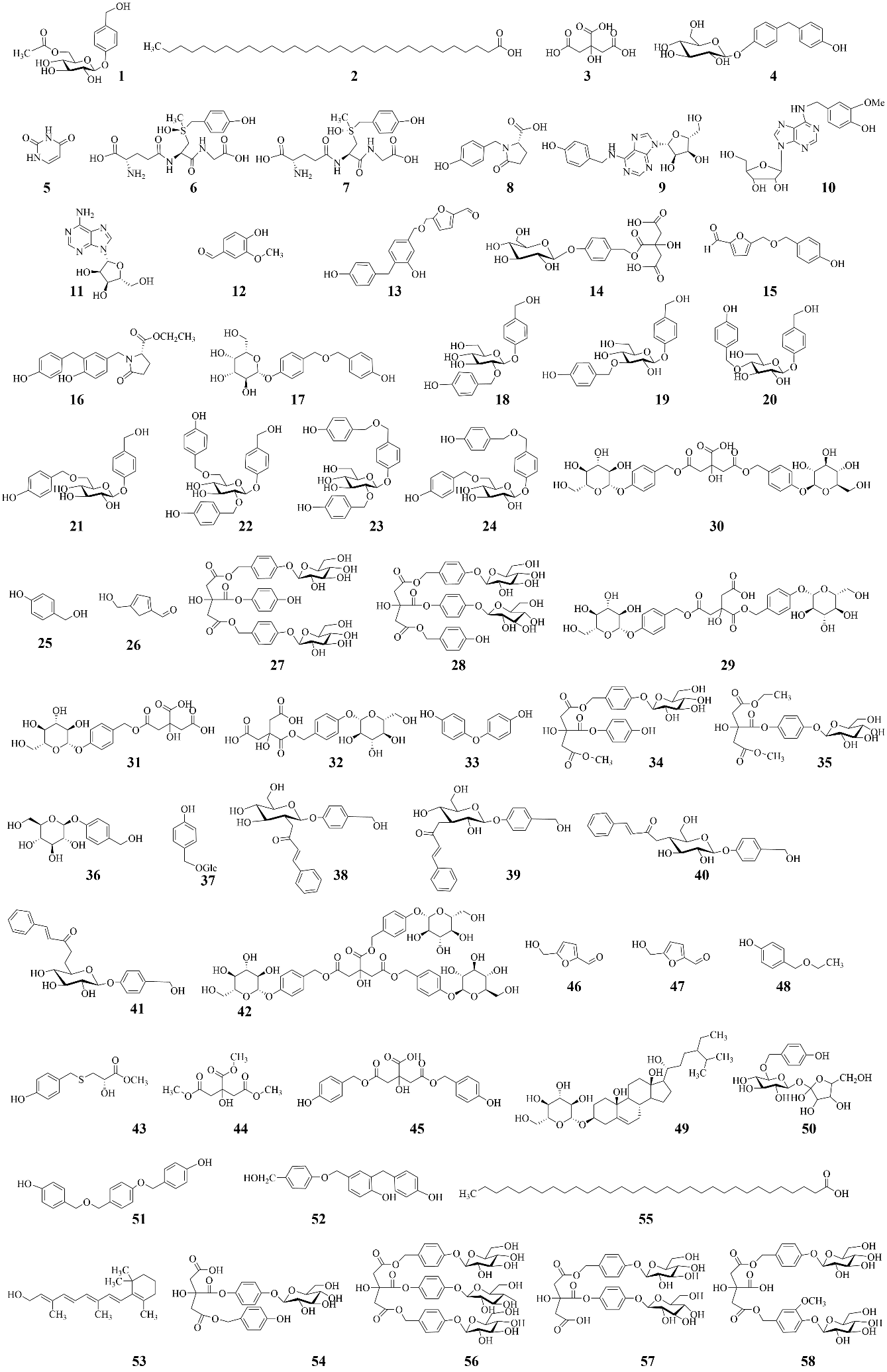


**Supplementary Figure S2.** Component structures obtained by UPLC-Q-TOF/MS for water extract of *Gastrodia elata* (WGE) identification*.*

## Supplementary Tables

**Supplementary Table S1** The qRT-PCR primer of cDNA fragment.

| Target gene | Forward primer (5’-3’) | Reverse primer (5’-3’) |
| --- | --- | --- |
| OPG | TGTTCTGGTGGACAGTTTGC | GCTGGAAAGTTTGCTCTTGC |
| RANKL | TCGGGTTCCCATAAAGTCAG | CTTGGGATTTTGATGCTGGT |
| Runx2 | CGAAATGCCTCTGCTGTTAT | TTCTGTCTGTGCCTTCTTGG |
| β-actin | CGTTGACATCCGTAAAGAC | TAGGAGCCAGGGCAGTA |
